# PReS-FINAL-1016: Micro vesicles as a magnifying glass; uncovering potential biomarkers in juvenile idiopathic arthritis

**DOI:** 10.1186/1546-0096-11-S2-P13

**Published:** 2013-12-05

**Authors:** G Keustermans, B Prakken, W de Jager

**Affiliations:** 1 Pediatrics, University Utrecht medical center, Utrecht, The Netherlands

## Introduction

Juvenile idiopathic arthritis (JIA) is a common chronic inflammatory diseases in childhood. Despite remission as a result of a plethora of treatment techniques, the chronic and relapsing nature of the disease requires continuous treatment which causes adverse side effects. It is important to uncover a biomarker that can efficiently predict patient responses to therapy as well as determine if patients will progress or regress as a result of treatment. Micro vesicles are key messengers containing many immune signaling molecules including cytokines, molecules known to play a major role in JIA.

## Objectives

Due to the localized inflammation seen in JIA, we aim to analyze if micro vesicles isolated from patients can provide a source of biomarkers, giving specific information on molecules that can be targeted for treatment and allow the disease state to be monitored.

## Methods

Micro vesicles where isolated from the blood and synovial fluid of patients with various subtypes of JIA. Vesicular protein profiles where then compared using Luminex technology.

## Results

Pilot data showed that whole JIA patient plasma and synovial fluid has an inflammatory phenotype expressing high levels of TNF-R1, S100 A12, CXCL9 and CXCL10. This phenotype is also seen in exoquick isolated plasma micro vesicles however, when micro vesicles are isolated by ultra-centrifugation, this phenomenon disappears. Ultra-centrifugation isolated vesicles express lower levels of IL-6, MIF, TNF-R1, CXCL9 and S100 A12 when compared to whole plasma and healthy control vesicles. An analysis of exoquick background activity on Luminex MIA technology reveals a high level of interference.

## Conclusion

Preliminary data indicates that micro vesicles isolated from JIA patient plasma by ultracentrifugation have low amounts of inflammatory cytokines. In addition, a more in depth investigation into exoquick activity shows that this product interferes with Luminex MIA technology. As a whole data seems to suggest that micro vesicle cytokine levels from individuals with JIA do not reflect the inflammatory process.

## Disclosure of interest

None declared.

